# Finding more constructive ways forward in the debate over vaccines with increased disability cultural competence

**DOI:** 10.1136/medhum-2021-012342

**Published:** 2022-04-29

**Authors:** Carolin Ahlvik-Harju

**Affiliations:** Theology, Åbo Akademi University Faculty of Arts Psychology and Theology, Turku, Finland

**Keywords:** medical humanities, medical ethics/bioethics, philosophy of medicine/health care, science communication, public health

## Abstract

The aim of this article is to study the discursive construction of disability that takes place in the vaccine-autism controversy from the 1990s to 2000s, and an attempt to develop a more holistic framework to understand vaccine decisions and their motivations. It is argued that the debate over vaccines produces knowledge and meanings about disability, and that the vaccine-autism controversy is kept alive largely because of how it reproduces stigmatising accounts of disability and autism. The suggestion is that if the stigmatising elements of disability were removed in the debate over vaccines, there would be no controversy to keep alive in the broader vaccine debate. Hence, this article is an attempt to increase disability cultural competence in the media and among health authorities and health professionals and therethrough broaden the shared understanding of what it means to be or become disabled. By investigating the driving forces for past vaccine controversies, the goal is to find more constructive ways forward in present day and future debates over vaccines.

## Introduction

Due to the COVID-19 pandemic, the world is in a state of global health emergency, and amidst this crisis, the development of vaccines has come to represent hope and the promise of going back to ‘normal’ in a not-too-distant future. But the fast development and rapid distribution of vaccines against COVID-19 have also caused anxiety and an ever so heated debate over vaccines. Conspiracies are widely spread (Soveri et al. 2021) and past controversies have gained new attention (Pullan and Dey 2021). For instance, the thalidomide scandal from the 1950s (Coombes 2016), the pertussis vaccine scare from the 1970s (Millward 2017), the vaccine-autism controversy from the 1990s to 2000s (Kaufman 2010) and the Pandemrix scandal in 2009–2010 (Oikkonen 2022), all represent past concerns related to the safety of vaccines, trust in authorities, risk evaluation, personal health versus public health, bioethics and disability; but they also have the power to affect vaccine uptake and vaccine attitudes in the present (Ashworth et al. 2021; Karlsson et al. 2021; Pullan and Dey 2021), as well as present-day illness narratives and the public debate over vaccines.

For instance, the intense debate over a possible link between the measles, mumps and rubella (MMR) vaccine and autism at the shift of the millennium provides a good example of risk evaluation in connection to vaccination, and the meaning-making potential in elaborations of a specific illness, in that case measles and autism. The vaccine-autism controversy lingers on in concurrent antivaccination rhetoric, and while the scientific understanding of autism aetiology has both changed and been disputed over the years, there are certain morally problematic understandings of autism that appear to have a strong hold in culture and society. These typically express stigmatising accounts of disabilities in general, and sometimes autism in particular (Kaufman 2010; Yudell et al. 2013).

While media coverage about the vaccine-autism controversy has been shown to be problematic, it has not been properly addressed in what ways (Conis 2016; Mann 2019). Since autism is at the very centre of the controversy, it is noteworthy that there is little research on the matter from a distinct disability perspective. One exception is, though, health communication scholar Benjamin Mann, who claims that autism narratives in media coverage of the vaccine-autism controversy may advocate for the elimination of autism (Mann 2019). This claim is further supported by autism advocate and writer Sarah Kurchak as she writes:

[---] that’s really what all of this is about: we’re facing a massive public health crisis because a disturbing number of people believe that autism is worse than illness or death. My neurology is the boogieman behind a completely preventable plague in the making (Kurchak 2015).

Mann and Kurchak highlight that the vaccine-autism controversy and the effects of it can be shown to be pertained with ableism, which is the belief that undesirable bodies can be separated from desirable bodies. Ableism is a cultural narrative that attributes disabled people the social status of less human. Ableism is the performance of inequality, injustice and discrimination against disabled people . Consequently, when autism, or disability in general, is being portrayed as the worst thing that can happen, it affects people’s vaccine attitudes and behaviour, and it continues a long and troublesome history of ableism. The implications for public health, for bioethics and for the families affected with autism are thus significant, and the value judgements involved in this discursive arena are salient, politically controversial and ethically questionable (Yudell et al. 2013).

The aim in this article is thus to study *the discursive construction of disability* that takes place in the vaccine-autism controversy, but it is also a contribution to the *development of more holistic frameworks to understand vaccine decisions and their motivations* (Carrion 2018). I argue that the debate over vaccines produces knowledge and meanings about disability, and that the vaccine-autism controversy is kept alive largely because of how it reproduces stigmatising accounts of disability and autism. That is: I suggest that if the stigmatising elements of disability were removed in the debate over vaccines, there would be no controversy to keep alive in the broader antivaccination movement. Therefore, this article is an attempt to increase disability cultural competence in the media and among health authorities and health professionals and therethrough broaden the shared understanding of what it means to be or become disabled. By investigating the driving forces for past vaccine controversies, I want to find more constructive ways forward in present-day and future debates over vaccines.

The outline of the article is designed as follows: first, I will give a more thorough background to what the vaccine-autism controversy was about, and explicate why its legacy is so relevant today. Second, I will discuss common values attached to disabilities and propose a rebranding of the normative, although problematic, assumptions about disability and autism that I find necessary for my constructive argument. Third, I will more specifically demonstrate how ableism plays out in the context of the vaccine-autism controversy, and point at the moral logic this enables. Fourth, I will explicate the theoretical premises and guiding principles for the account of disability cultural competence that I propose and discuss the concrete knowledge areas involved. Lastly, I will draw together the conclusions of the article. Since the emphasis here is on the performance of ableism and on the moral logic this enables in the context of vaccines, this article is theoretically and methodologically rooted primarily in (bio)ethics and disability theory. Most noteworthy are the works of Rosemarie Garland-Thomson whose writings on disability cultural competence are central to my argument, and Alison Kafer whose work Benjamin Mann uses in his analysis. The conclusions are, however, a contribution to the growing field of qualitative and philosophical research on vaccine attitudes, and health communication in a broad sense.

### The vaccine-autism controversy

By the early 21st century, the idea that something about childhood vaccinations are the cause, may be the cause, or may be one contributing factor in the apparently rising numbers of children with neurodevelopmental problems had spread widely among U.S. (and other) parents (Kaufman 2010, 9).

Sometime around the turn of the millennium, the story of a connection between vaccines and autism started to emerge in the media. Vaccine safety and vaccine risks had naturally been discussed before that, but around the year 2000 reports about vaccine dangers became a more regular feature in the news (Conis 2014). Many claim that the main reason for this was the Andrew Wakefield study from 1998—which suggested a possible link between the MMR vaccine and autism—but the fact is that vaccine-safety activists had long argued that immunisation has the potential to affect the immune and nervous systems in ways that could cause autism in some cases. Not least in the 1970s and 1980s after the pertussis vaccine scare, which started with a group of British parents claiming that their children had become disabled as a result of the whooping cough vaccine (Millward 2017). Nevertheless, in the aftermath of Wakefield’s study, parents, doctors, health experts and journalists started to examine the vaccine-autism connection more eagerly than before. In fact, only in the USA funding grants climbed by millions each year between 2000 and 2011, and although a vast number of studies conclude that there is no causal link between vaccines and autism (Navin 2017), the narrative of its possibility is kept alive in the media and among parents and health professionals (Conis 2014; Silberman 2015).

One reason for this is that although vaccine worries were not new at end of the 1990s, widespread fear of autism was. When in the 80s, autism affected 1 in 10 000 children, the constantly growing number was 1 in 500 children around the year of 2000, and 1 in 68 children by 2015. The condition appeared to spread like an epidemic, and despite research suggesting otherwise, many parents suspected vaccines to be the main cause for the rise. Studies on health communication show that during this time stories about children living with autism started to appear more frequently in the media, and a common feature of these stories was parents speculating on whether the autism was brought on by vaccines or not. Hence, at the end of the century vaccine worries were commonly made part of articles about autism, and autism worries started to appear in official reports on vaccines and vaccine policy (Blume 2017; Conis 2014; Mann 2019).

While the news media did incorporate vaccine worries stories, the internet became the main platform for parents to circulate their suspicions. Parents to children with autism—who oftentimes had been struggling with getting help and sympathy from healthcare professionals—now had a huge community of parents to share their worries with, and a huge experiential knowledge base about their children’s condition. And although these internet-based communities gave parents a highly longed for support, they also contributed to a divide between families with autism and health professionals (Brock 2019; Conis 2016), particularly between mothers and health professionals. The mothers are usually the ones more responsible for making decisions about medical interventions for their children, and in political, popular and scientific discourses about vaccinations, ‘parental’ responsibility has typically meant ‘maternal’ responsibility (Conis 2016; Navin 2017). Historian Elena Conis shows that vaccination campaigns from the 1950s through the 1970s had routinely emphasised maternal responsibility for obtaining needed vaccines for children (Conis 2016), and although the effects of such rhetoric are complex, what is relevant in the context of the vaccine-autism controversy is that mothers have long been blamed for the poor health status of their children. Early research on autism suggests, for instance, that autism was a result of a dysfunctional early attachment between mother and child (Navin 2017). Hence, the urge to find all information out there and do as much as possible to ensure the safety of one’s child needs to be understood against the cultural development of parenthood as a fundamentally moral act (Ahlvik-Harju 2019; De Benedictis 2012). It is however important to recognise that media representations of the autism epidemic pitted mothers against experts and institutions that did not listen to them. This enabled autism advocacy to turn into vaccine-safety activism (Conis 2016).

The vaccine-autism controversy affected the MMR-vaccination rates negatively in many countries around Europe and in North America, but it is interesting to note that there are exceptions that might be connected both to the way media has reported on the issue and to the way health authorities and governments have handled the controversy. In the UK, the vaccination rates for the MMR vaccine dropped from 92% to 80%, as the vaccine-autism controversy took centre stage in the broader political discourse about policy and science at the time, and the vaccine debate was made part of the national news agenda (Stöckl and Smajdor 2017). In Finland, on the other hand, the vaccination rates did not drop dramatically around the time of the controversy and is therefore somewhat an outlier in the research on international autism media coverage. A recent study (Pesonen et al. 2020) suggests that some uniquely Finnish cultural norms may have played a role in how newspapers approached the vaccine-autism controversy and autism as a condition.

The damage of the vaccine-autism controversy to public health continues, and it is clearly fuelled by unbalanced media reporting, and by an ineffective response from governments, researchers, journals and health professionals (Blume 2017). On a deeper level, the vaccine-autism controversy has never been merely about science or medicine. It was and is also about values related to risk-evaluation and illnesses/disabilities (Conis 2016). Although seldom discussed, this phenomenon is further exemplified in the ‘re-branding’ of illnesses that can be found in histories of vaccination, that is, the conscious efforts by health authorities and politicians to encourage fear of a disease in the face of introducing a vaccine (Blume 2017). Hence, the values attached to various illnesses and disabilities are an important part of the vaccine debate and the broader discourse about public health.

In the following section, I will discuss the values attached to disabilities and specifically autism, since I stress that the account of disability cultural competence that I am proposing, demands a rebranding of normative assumptions about disability and autism.

### What is disability? What is autism?

Somehow a way must be found to wrench the idea of disability out of its etymological linkage with lack and brokenness (Brock 2019, 171)

In this article, I use the term autism to describe the condition in a very broad sense, while I recognise the problematic nature of such generalisation. The choice to do so is partly due to the fact that there is no uniform stance on what language to use, even in the autism community itself, that is, autistic people, their family and friends as well as their broader support network do not agree on how to describe autism. Neither do various disability rights groups, the neurodiversity movement, nor the scientific community agree (Kenny et al. 2016). However, as will become apparent in the ‘Ableism in the context of the vaccine-autism controversy’ section, the media has oftentimes invoked stereotypical and stigmatising accounts of autism when reporting on the vaccine-autism controversy (Mann 2019). For this reason, it is important to recognise that the language used has the power to reflect and shape people’s perceptions of autism. Hence, the choice to speak of autistic people instead of people with autism derives from the stance that there is nothing inherently negative about being autistic. This autism-affirmative language is preferred by many adults with autism (Kenny et al. 2016), and is much aligned with the premises and purposes of this article.

In what follows I will, however, discuss the problematic—although established—understandings of disability and autism and state the perspective on disability that sets the foundation for my constructive argument. The guiding question is: what would it mean to place autism in the context of disability, and what account of disability would be helpful in the shaping of an autism-inclusive vaccine debate?

There are many different accounts of disability and various models have varied a lot between countries and cultural contexts, but the growing literature in disability studies has continuously brought forth critical analyses and proposed new understandings (Ahlvik-Harju (2016)). Nevertheless, the persistent dominant view is that disability implies pain, suffering, disease, functional limitation, dependence, social stigma and a life with limited opportunities. Consequently, the birth of a child with disability or the onset of disability is usually seen as a catastrophe. To enter into the cultural category of disabled is, then, not simply to be looked on as sick, but to endure sufferings and exclusions and isolation distinct from other marginalised identity groups (Garland-Thomson 2012).

When speaking of autism, it refers to a broad range of behavioural, language and communication deficits, and issues related to neurodevelopmental dysfunction. In the diagnostic world, autism encompasses a range of symptoms labelled autism spectrum disorders, which include autistic disorder, Asperger’s disorder or syndrome and pervasive developmental disorder, not otherwise specified. The diagnoses might sound clear, but the fact is that they are not clear at all, and different doctors might evaluate the symptoms in totally different ways. This is the issue with conditions located in the mind: the symptoms are not easily defined, and they may change over time. Therefore, autism represents a labelling process and a category of knowledge in which the boundaries of symptom inclusion and exclusion are fluid (Brock 2019; Kaufman 2010).

Because of the unspecific nature of the condition, autism has come to be the go-to-diagnosis when neurodevelopmental dysfunctions occur in a child. Research indicates that the prevalence of children with autism spectrum disorder has risen dramatically, but mutable disease classification and disease recognition are both central to the observed increase in prevalence rates. Although some disagree, most epidemiologists and clinicians agree that the expanded diagnostic categorisation, greater parental awareness, more refined testing for children, more health professionals trained in diagnosis and referrals for diagnosis and a growing demand for services that can be accessed only through a diagnosis, all contribute to an increased rate of diagnosis (Kaufman 2010). This is an important point with regard to the vaccine-autism controversy and the whole argument of how autism has become an epidemic.

Besides these points, there are also other more complex aspects of the categorisation of autism that need to be mentioned because they highlight power relations so relevant to any account of disability—and in this case autism. Heilker (2012) views autism as a profoundly rhetorical phenomenon. He states:

We do not yet know what causes autism, and outside of any personal experiences we have had with autistics, all there is to work with is a fundamentally uncertain yet aggressively suasive public discourse about the condition, where there is heated argument about what, exactly, autism is, how we should think about it, and how we should respond to it (Heilker 2012).

Heilker points at the discursive element of autism which does not necessarily have truth as its goal, but to create uncertainty by competing narratives. Since most people do not have regular, personal contact with autistic individuals, people’s conceptions of autism and autistics are driven primarily by what they encounter in popular media and by the aggressive public discourse about the condition. He points out that autism in large appears to be a white phenomenon, and it is interesting to note that, in the USA, several autism advocacy groups who largely tap into antivaccination rhetoric as well, are fronted exclusively by white celebrities. But autism as a white phenomenon turns out not to be merely discursive, because there are significant disparities in access to healthcare when comparing Hispanic communities and people of colour with the white population, which highlight important societal injustices with regard to health in general, but they also hide possible autism diagnoses among these populations (Bishop-Fitzpatrick and Kind 2017; Conis 2016; Heilker 2012; Mandell et al. 2002).

A racialised perspective on autism is thus important, because without it, the disability perspective runs the risk of hiding coloured experiences of autism too. To understand autism from the horizon of disability does necessitate taking serious the various ways in which race is a part of the performance of disability (Kafer 2013; Puar 2017). Furthermore, studies show that vaccine resistance is closely connected to wealth and to the collective perception of risk and its relation to social inequality and solidarity (Berezin and Eads 2016), which highlights that a power perspective is especially pressing when discussing autism in the context of the vaccine-autism controversy.

What many autism community members underline is this: “if you know one person with autism, you know *one* person with autism”. Autism is a many-dimensional manifold of abilities and limitations. Therefore, it might be worthwhile following autism researcher Ian Hacking’s advice and speak of autistic communities in the plural (Hacking 2010), and so join the neurodiversity movement claims that there are neurological differences in the human population, and that autism is a natural variation among humans—not a disease or a disorder, just ‘a difference’ (Runswick-Cole 2014). But somehow the diversity card does not seem to be all that helpful since it ignores some of the hardships that are affirmed in the lives of many disabled people (Ahlvik-Harju 2016). Instead, disability could be thought of as something that begins in bodily variation, but transformed by encountering the world. Rosemarie Garland-Thomson writes:

The human variations we think of as disability are interruptions of departures from a standard script of human form, function, behaviour, or perception of what we call normal. Disability, then, is the transformation of the flesh as it encounters the world. Disability is the response over time to its environment. Disability occurs when the shape and function of bodies come into conflict with the shape and stuff of the world. The discrepancy between body and world, between that which is expected and that which is, produces disability as a way of being in an environment. So whereas disability is an index of capability in context, it is also a witness to our receptiveness to being shaped by our life journeys in the world (Garland-Thomson 2012, 342).

One argument in this article derives from this particular understanding of disability, namely that autism is something that begins in the mind of an individual but transforms into disability as the autistic individual is shaped in and by the world. I argue that, when framed this way, the complexities around autism become easier to respond to. This implies that disability is inherent in human being and therefore it is even more urgent to critically assess contemporary discourses that approach disability in ways that inevitably shape common understandings of both disability and people’s sense of selves (Garland-Thomson 2012).

A final note about how to understand disability that is important for the constructive argument is that disability is inherent in the human condition *now*—disability is something to hold on to for the *future*. Garland-Thomson uses the word conserve in this context. The idea of holding on to or conserving suggests that the characteristics that are thought of as disabilities should be understood as benefits rather than deficits, that is, disability is more of a resource than a liability. Although the collective consciousness denies characteristics such as vulnerability and mortality, disability insists that people’s bodies are dynamic. Bodies need care; bodies are fragile; bodies are limited; bodies need assistance to live. Hence, disability is not a resource in an economic sense, but disability is a resource in that its presence in the world works as a reminder of the giftedness of life, and of the fact that life cannot be fully controlled (Garland-Thomson 2012).

### Ableism in the context of the vaccine-autism controversy

As mentioned, there is little research on the vaccine-autism controversy from a distinct disability perspective. A welcome exception is however a study by Mann (2019), in which he focuses on how narratives about the vaccine-autism controversy may seek to eliminate disability. Drawing on the important work of disability scholar Alison Kafer, Mann analyses media coverage from the 10 most popular online news sources in the USA, and he found three major narrative themes: the death and survival narrative, the societal problem narrative and the preventative narrative. The results of this study reflect the general trend in the vaccine debate and is therefore—I suggest—a good demonstration of how ableism plays out in the immunisation context. In this section, I will present some of the ableist rhetoric that occurs in the vaccine debate.

Initially, a few things can be said about the problematic features in news articles about autism. There is, for example, a clear tendency to discuss approaches to autism in terms of medical interventions, which serves to exemplify how medicalisation of autism is well established. It is also found that autistic children are portrayed more frequently than autistic adults, which is a way of infantilising autism. Furthermore, in many news articles about autism there is no clear definition of what autism is, leaving the reader with stereotypical images (Mann 2019). These are examples of how autism is narrated according to an ableist logic to medicalise, belittle and diminish the condition. Because autism is medicalised, the widely accepted way of framing disability in the Western world is with the medical model (Davis 2013; Grue 2017). The infantilisation of disability is common practice in other contexts as well, and further reinforced in relation to gender (Malacrida 2009; Robey, Beckley, and Kirschner 2006).

Mann suggests that the *death and survival narrative* in vaccine-autism news laments the status of autistic children as a sort of death or loss that denies them a possible future, while also pushing the child and family to survive the diagnosis. What is problematic here is to place death on the autistic child, while describing the family as the survivor (Mann 2019). Although grief is in fact one of the most common parental emotions on diagnosis (Rabba, Dissanayake, and Barbaro 2019), it is crucial to separate the personal feelings that occur in the landscape of parenthood, and what is made the accustomed attitude towards autism in general. Because as Brian Brock, father of an autistic son, withholds: grief is only one emotion that predominates in loving someone with autism (Brock 2019). Furthermore, the death and survival narrative also include how autism is portrayed as a disease that invade the family, making autism an external threat of some kind. Once again, it is important to recognise that autism—in all its various expressions—does affect the autistic child and the whole family. For instance, Myers, Mackintosh, and Goin-Kochel (2009) give a rich account of how the whole family might experience a child with autism (Myers, Mackintosh, and Goin-Kochel 2009), but the risk in framing autism as something external is placing the autistic child outside the family unit in some sense. Put together with statements like “I would rather have a child dying from measles than being sentenced to a lifetime of autism” (Mann 2019), stigmatisation is a fact.

*The societal problem narrative* further suggests that autism is a personal death and a societal one. In the immunisation context, it is not unusual to express worry about the rising numbers of children with autism, followed by a comment about how this is going to destroy the world or be disastrous to society. The implication of this is that autism is perceived as a problem to be dealt with in the present if the future is to be saved (Mann 2019). Andrew Wakefield and Del Bigtree, who are two key figures in today’s antivaccination movement in the USA and beyond, frequently stress that the rising number of autism is going to be the end of society. Hence, the societal problem narrative is truly at the core of present-day antivaccination rhetoric (Wakefield 2016).

*The preventative narrative* represents examples of when autism is discussed as something to be prevented. Here, prevention is synonymous to the desire for a future without autism (Mann 2019). Many articles speak to medical solutions that may prevent autism entirely, thus fitting well into the goal of curing rather than caring common in modern medicine. This is an occurring phenomenon in other medical contexts as well, but not only in a rhetorical manner. The introduction of prenatal testing, for instance, was motivated on the grounds of prevention. That is prevention from future costs and prevention from suffering, an elaborate way of saying that children with disabilities are not wanted (Ahlvik-Harju 2014). In the immunisation context, this is further exemplified in articles that aim to debunk misinformation about the vaccine-autism link with possible ways to reduce autism in the future (Mann 2019).

When thinking about ableism and ableist rhetoric, what presents itself in the context of immunisation is clearly a medicalised, functionalised, racialised and capitalised view of human being(s). Medicine is to solve all kinds of human challenges and problems, human existence is defined by certain abilities and functions, the only significant human experience is white and human beings must not be an economic burden to families or societies. And while most people know that able-bodiedness is only temporary (because life is finite) and that physical and mental impairment can occur at any time, much of the physical, social and cultural world is designed in ways that discriminate against people with disabilities. The strength in Mann’s analyses lies here in the theoretical framework of Kafer (Kafer 2013). Namely, her activist approach that politicises stigmatising language about disability and her envisioning of a future with disabilities rather than the opposite that so evidently occurs in antivaccination rhetoric is what makes Mann’s examples stand out.

While ableism is not merely a rhetorical problem, the vaccine-autism controversy does demonstrate the power of language and rhetoric. Therefore, any means of challenging ableism must confront its rhetorical power. When discriminative rhetoric becomes so widely reified as autism has become in the immunisation context, it needs to be properly addressed and stripped of its impact (Cherney 2011). In the ‘Disability cultural competence’ section, I will present Garland-Thomson’s practice disability cultural competence, which sets the foundation for my constructive argument about how to find a more constructive way forward in the debate over vaccines.

### Disability cultural competence

In the articles ‘Disability Bioethics: From Theory to Practice’ (2017) and ‘Disability Cultural Competence for All as a Model’ (2021), Garland-Thomson (the latter co-written with Lisa I. Iezzoni) puts forward an initial proposal for a practice called disability cultural competence. The goal of this practice is broadly to shape attitudes, policy and practice (Garland-Thomson 2017) according to the view that disability is something worth preserving or conserving (Garland-Thomson 2012), which is aligned with Kafer’s view presented in the ‘Ableism in the context of the vaccine-autism controversy’ section. This view of disability and human being is crucial because it sets this specific account of disability cultural competence apart from many other accounts, in that it does not reflect a thin understanding of disability culture and disability equity. That is, its goal is to establish a sense of pride and positivity as opposed to mere acceptance or tolerance. Its goal is to develop competencies to use the world effectively, maintain dignity, exercise self-determination, use accessible technology, find community and maintain relationships. Its goal is to bring disability culture to people currently identified as disabled and their families and caregivers as well as to people who might become disabled in the future. In question is also a form of ‘structural competency’, because its attention is focused on how social and cultural structures influence health outcomes and shape personhood beyond individual collaborations (Garland-Thomson 2017). In what follows, I will explicate the theoretical premises and guiding principles for disability cultural competence and discuss the concrete knowledge areas involved.

The theoretical premises for disability cultural competence is found in two bioethical works, namely in the writings of bioethicist Jackie Leach Scully who argues that a *distinctive moral knowledge* can arise from the experience of living with a disabled body, and in the *disability principlism* articulated by law professor Alicia Ouellette. The idea behind the first claim is that people draw on their bodily experiences as they think and construct their social reality. The body is with its form, functions and perceptual anxieties—in this perspective—the horizon from which the world is understood. Thereby the experience of being a minority living in a world built for the majority can open new moral understandings. The idea behind disability principlism are the principles of: non-discrimination; full and effective participation of people with disabilities in society; respect for difference and accessibility (Garland-Thomson 2017; Garland-Thomson and Iezzoni 2021). Garland-Thomson argues that a disability cultural competence based in these premises strengthens the cultural, political and institutional climate in such a way that people with disabilities can flourish just as they are, and I further believe that this form of disability cultural competence could be a game changer in the vaccine debate. Although Garland-Thomson recognises that much has changed with increased political rights for people with disabilities, she stresses:

[-] all of us sense that the old way of talking about disability as a curse, tragedy, misfortune, or individual failing is no longer appropriate in our post-disability rights era but we are unsure about what more progressive, less impolite, language to use. Because of this, we are often reluctant to recognize our fellow citizens as disabled people, and often even more reluctant to acknowledge our own experience or status as people with disabilities (Garland-Thomson 2017, 333).

The long history of devaluing disability is deeply embedded in language, and in the previous sections, I have demonstrated how this is manifested in the vaccine debate. Ableism is in large a rhetorical problem because ableism sustains and maintains itself via rhetoric (Cherney 2011) and therefore a crucial element of disability cultural competence is proposing language and ways of talking about disability that are non-prejudicial; a language that does not communicate stigma or tragedy; a language that does not impose failure of normalcy (Garland-Thomson 2017). Appropriate language is key in the process of unlearning attitudes that assign people with disabilities the role of the unwanted or the ultimate ‘other’ (Ahlvik-Harju 2015), but not the only focus.

Disability cultural competence is intended to be a practical toolkit that identifies and develops supports for people with disabilities and it involves five areas of knowledge: (1) biomedical decision-making, (2) disability culture and history, (3) accessible technology and design, (4) disability legislation and social justice and (5) disability cultural competence research. Hence, crucial to disability cultural competence is awareness about, support for and promotion of disability history, culture and arts. It is about user-based design, development and promotion of accessible technology that helps improve the quality of life for people with disabilities. It is about knowing the legal rights, obligations and protections due to those identifying as people with disabilities (Garland-Thomson 2017; Garland-Thomson and Iezzoni 2021).

The foundation for these knowledge areas is the disability bioethics presented earlier, which ought to be translated into a specific set of principles, practices, policies and competencies that can affect biomedical decision-making and life decision-making. While Garland-Thomson underlines that disability cultural competency would begin in healthcare environments, and from there extend to workplaces, commercials, governments, culture and private organisations (Garland-Thomson 2017), I would propose a more horizontal and synchronised endeavour.

The bioethical framework is important since bioethics springs from the need for a response to unethical medical practice (Garland-Thomson 2017). And while ableism exists in every sphere of society and culture, the medical sphere has been the dominant context with regard to ableist definitions of and responses to disability. Elsewhere I have emphasised that health professionals do not merely offer support to people with disabilities. They also possess the power to act as gatekeepers of treatment, social benefits and inclusion. Hence, they influence both health policy and attitudes towards health in general. They have the power to control language, knowledge and even the social response to disability, and therefore they play a crucial role in maintaining and justifying the view that disability is an individual tragedy (Ahlvik-Harju 2017).

Because health professionals have been and are in an obvious power position in relation to people with disability, a distinct disability bioethics that frames disability as valued social diversity, and supports the human rights-based understanding of disability in legislation, can move both disability and bioethics out of the healthcare context and into other material environments, civic institutions, cultural structures and interpersonal interactions. Thus, disability cultural competence is a skillset that springs from bioethics and biomedical decision-making in the healthcare setting, but reaches towards everyone as they navigate through life in egalitarian democratic societies (Garland-Thomson 2017).

The tools of disability cultural competence would be courses, training, presentations, expert patients, certification, speakers, exhibits and media products. Garland-Thomson suggests that the primary leadership in disability cultural competence development and implementation would come from expert communities in disability bioethics and from subject experts in disability culture. Leaders and tool developers would be people with a high degree of disability cultural skill. A disability cultural competence initiative would produce research, policy papers, events, education, curation and support for disability cultural competence implementation (Garland-Thomson 2017).

The question is, then, in what way the vaccine-autism controversy and the wider debate about vaccines could change if disability cultural competence would be implemented? That will be discussed in the ‘Concluding discussion’ section.

## Concluding discussion

Many want to blame Andrew Wakefield and the antivaccination movement for declining vaccination rates and increased illness outbreaks, and some want to put the responsibility on the media. Not for the doubts about vaccine safety that the vaccine-autism controversy has caused necessarily, but for the narrow and simplistic explanations available to worrying parents (Blume 2017; Conis 2014). Placing the responsibility solely or primarily on the antivaccination movement (or Wakefield) ignores other factors to why people choose not to vaccinate, and it ignores the specific context(s) in which the controversy has gained a strong hold. It ignores deeply held values that health and risk communication both reflect and generate as knowledge is circulated between parties. Therefore, I argue that it is important to investigate the driving forces for past vaccine controversies.

In this article, I have demonstrated that the vaccine-autism controversy is not merely a medical problem or an epidemiological problem. I have shown that it in large is a diagnostic problem, but most importantly I have wanted to show in what ways it has turned into a *moral problem*. It is a moral problem because, at its core, the vaccine-autism controversy is about what kind of human beings that are welcomed into this world—and what kind of world that is built for human beings already inhabiting it.

I have demonstrated that the vaccine-autism controversy is pertained with ableism by presenting different narratives about disability that make up an important part of the discourses about a possible link between vaccines and autism. These narratives say that autism denies people a happy future. They say that families need to fight an enemy that comes in the form of an autism diagnosis. They say that the rising number of autism is going to be the end of society. They say that as much preventive work as possible is necessary to eliminate autism, because if it is not done, then families and society will suffer from economic and social costs. Ableism is by no means only a rhetorical problem, but the vaccine-autism controversy does demonstrate the power of language and rhetoric. Therefore, any means of challenging ableism must confront its rhetorical power.

Discursively the controversy expresses and upholds stereotypes of disability that, among other things, leads to people—particularly parents to young children—fearing neurological and behavioural disabilities. Because parenthood is understood to be a moral act, the discursive construction of disability can be described as the performance of ableism. In line with Garland-Thomson, I therefore argue that the complex experiences of autistic people should lead future conversations about autism. A crucial element of disability cultural competence is proposing language and ways of talking about disability that are non-prejudicial, that does not communicate stigma or tragedy, and that does not impose failure of normalcy. I find this to be necessary in all health communication. Health professionals, health authorities and the media (in all its various forms) have the power to shape attitudes in a very broad sense and can thus invite everyone into building solidarity with others and create a more open society—a more autism-inclusive society. To place the lived experience of autism as the horizon from which to guide the (moral) responses in the vaccine debate is a good starting point for challenging the rhetorical power of ableism. I truly believe that this could be a game changer in the debate over vaccines and for trust-building between families and health professionals. Furthermore, it would be a game changer for the 15% of the world’s population who identify as disabled (WHO 2021) to finally belong, and to be perceived as having a future.

The reason to why I argue for the implementation of disability cultural competence as opposed to merely focusing on challenging the rhetoric is related to the fact that health professionals are in an obvious power position in relation to people with disability, but also in a key position with regards to vaccine attitudes and behaviour. Therefore, I believe that a distinct disability bioethics that frames disability as valued social diversity and supports the human rights-based understanding of disability in legislation can move both disability and bioethics out of the healthcare context and into other material environments, civic institutions, cultural structures and interpersonal interactions. Hence, to work with disability cultural competence on a broader level is an attempt to connect concerns over vaccines with other concerns in the medical sphere. It is an attempt to connect policy and practice in a way that actually has the power to change the moral landscape in the medical context and in the debate over vaccines.

What if parents to young children would be allowed to circulate their worries at the doctor’s office and have someone say that autism might be a challenge, but also a gift? What if more research funding could go into creating the necessary support for autistic families, instead of merely to finding out *why* they have autism in the first place? What if representants for the media would highlight a more complex and optimistic view of (people with) autism when reporting on the safety of vaccines? What if the presence of the 1 in 68 people (or more) that are diagnosed with autism would be a reminder of life’s giftedness, rather than a sign of catastrophe? What if research about vaccine attitudes, public health and bioethics would extend its basic premises, principles and views on human being so that they would actively work against polarisation and discrimination of people with disabilities?

To find language, forms and norms for that which here is called disability cultural competence is about finding more human ways of perceiving people labelled disabled, and about finding concrete tools to in more sensible, truthful, constructive and competent manner discuss vaccines and autism. Because when both research and the wider discourse are limited to whether there is a link between the MMR vaccine and autism, there is an implicit ableist message there that autism is not wanted. People with autism are not wanted. Bearing in mind that there will be other pandemics and health emergencies in the future, there is a need for competencies to determine the difference between facts and myths, and there is a need for competencies to find common ground and common goals, competences that link tender discursive skills with compassionate practices, competence to recognise the moral challenges in the medical sphere. Implementing disability cultural competence has the generative power to do just that.

COVID-19 has presented many challenges with regard to illness narratives, risk-evaluation, trust in authorities and vaccination. Although this article is focused on the vaccine-autism controversy, my hope is that the outcomes of it can have bearing on present-day vaccine debates and on present-day healthcare practices. For instance, in what ways can the performance of ableism be discerned in the media coverage of COVID-19? In what ways can ableism be discerned in access to healthcare, in administration of vaccines and in COVID-19-related antivaccination rhetoric? And with occurring reports on long COVID-19: what future can be discerned for all those lives affected long-term with COVID-19?

## Data Availability

No data are available.
